# Intra-atrial Conduction Block During Radiofrequency Ablation of Left-sided Accessory Pathways

**DOI:** 10.19102/icrm.2022.130205

**Published:** 2022-02-15

**Authors:** Dimitrios Asvestas, Stylianos Tzeis, Konstantinos Letsas, Christina Goga, Michael Efremidis, Panos Vardas

**Affiliations:** ^1^Cardiology Department, Mitera General Hospital, Hygeia Group, Athens, Greece; ^2^Second Department of Cardiology, Laboratory of Invasive Cardiac Electrophysiology, “Evangelismos” General Hospital of Athens, Athens, Greece

**Keywords:** Accessory pathway, atrioventricular reentrant tachycardia, catheter ablation, intra-atrial block, mitral isthmus ablation

## Abstract

Catheter ablation is currently the therapeutic approach of choice for many patients with accessory pathways. Despite the high success rate of radiofrequency ablation of the left lateral accessory pathways, a rather uncommon manifestation is intra-atrial conduction block at the level of the mitral isthmus. We report 2 cases of orthodromic atrioventricular reentrant tachycardia using a concealed left-sided accessory pathway with an abrupt change in the activation of the coronary sinus from an eccentric to concentric sequence after ablation delivery. The electrophysiological characteristics and the underlying mechanism of the intra-atrial conduction block are commented on. Careful mapping and assessment of relative conduction are helpful to document the diagnosis of intra-atrial conduction block. Familiarity with the likelihood of intra-atrial block during left lateral accessory pathway ablation is needed to avoid the erroneous elucidation that a second accessory pathway is present and to identify correctly the ablation site of interest.

## Introduction

Catheter ablation (CA) is currently the therapeutic approach of choice for patients with accessory pathways (APs). Despite the high success rate of CA in patients with a left lateral AP, a rather uncommon pertinent caveat is the intraprocedural occurrence of an intra-atrial conduction block at the mitral isthmus level.^1–4^ We present 2 cases of atrioventricular (AV) tachycardia due to a concealed left lateral AP, with an intra-atrial conduction block during radiofrequency (RF) CA.

## Patient 1

A 38-year-old woman with a history of supraventricular tachycardia was referred to our hospital for invasive management. The 12-lead electrocardiogram during sinus rhythm was normal. After obtaining signed informed consent from the patient, 2 quadripolar diagnostic catheters and 1 decapolar catheter were positioned via the right femoral access at the His, the right ventricular (RV) apex, and the coronary sinus (CS), respectively.

Programmed atrial stimulation showed decremental AV conduction without dual AV node physiology. During RV pacing, an eccentric atrial activation sequence with non-decremental conduction properties was documented. Following incremental ventricular pacing, a narrow QRS, short RP tachycardia with a 1:1 AV relationship was induced at a stable cycle length (CL) of 320 ms. Overdrive pacing from the RV apex at a CL 20 ms shorter than the tachycardia CL (TCL) revealed a V–A–H–V response upon pacing termination. The difference in the: post-pacing interval (PPI) minus TCL ruled out AV nodal re-entry tachycardia (AVNRT). Therefore, an orthodromic AV re-entrant tachycardia (ORT) via a left-sided AP was diagnosed.

**Figure 1:**
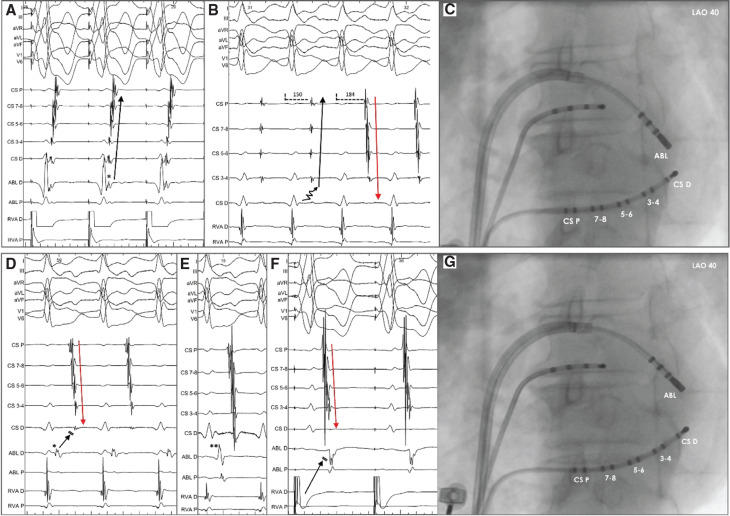
Ventricular pacing showing retrograde activation of the left atrium via a posterolateral accessory pathway—the atrial activation sequence is eccentric and the ablation catheter records a fused V–A electrogram (asterisk) **(A)**. During radiofrequency energy delivery, the V–A interval was prolonged initially at 150 ms with a distal to proximal activation pattern. Subsequent V–A prolongation (184 ms) with a concentric coronary sinus activation pattern (red arrow) was noted during ablation **(B)**. The prolongation of the V–A interval could be explained by the slow conduction (zig-zag arrow) along the mitral isthmus, while the reversal of the atrial sequence is associated with a mitral isthmus block. **C:** The position of the ablation catheter in a 40° Left anterior oblique projection is shown. In a more lateral location, a fused V–A interval is recorded at the ablation catheter, suggesting a target ablation site **(D)**. A longer V–A interval is recorded when the ablation catheter is moved to the septal mitral annulus (double asterisk in **E**). Radiofrequency application at the site with the shortest V–A interval (asterisk) led to the successful abolition of the accessory pathway. **F:** Postablation recording of a counterclockwise atrial activation sequence with a long V–A interval at the lateral mitral annulus during ventricular pacing. **G**: The fluoroscopic position of the ablation catheter in a 40° left anterior oblique projection at the site of accessory pathway elimination is shown. *Abbreviations*: ABL, ablation catheter; CS D, coronary sinus distal; CS P, coronary sinus proximal.

Subsequently, AP mapping was performed using an irrigated catheter via a transseptal approach. The earliest retrograde atrial activation was documented at the posterolateral mitral annulus (4 o’clock in the left anterior oblique [LAO] projection), distal to the 1-2 bipole of the CS catheter **(Figures 1A and 1C)**. Deployment of ablation lesions at this site resulted in the immediate termination of the tachycardia. However, the ORT was still induced reproducibly with a prolonged V–A interval. Meticulous mapping identified the site of earliest retrograde atrial activation at a slightly more anterior location, where additional lesions were delivered, resulting in the further prolongation of the V–A interval (from 150 to 184 ms) and reversal of the retrograde atrial activation sequence to a concentric pattern **(Figure 1B)**. The TCL remained unaffected. Interestingly, the V–A interval at the lateral mitral annulus, distal to the level of the deployed lesions, was shorter than the V–A recorded septally **(Figures 1D and 1E)**, despite the presence of a proximal-to-distal CS activation. These findings suggested an intra-atrial block at the posterolateral mitral annulus, proximal to the AP insertion, resulting in a counterclockwise propagation of the retrograde wavefront along the mitral annulus, with a concentric CS activation sequence. Additional RF delivery distally to the last lesions terminated the tachycardia. Post-ablation ventricular pacing demonstrated concentric retrograde atrial activation with decremental conduction properties. Furthermore, the ablation catheter positioned at the mitral annulus laterally to the CS catheter was activated later than the distal CS bipole **(Figure 1F)**. No tachycardia was inducible following atrial or ventricular pacing. The patient remained asymptomatic during a 4-month follow-up.

## Patient 2

**Figure 2:**
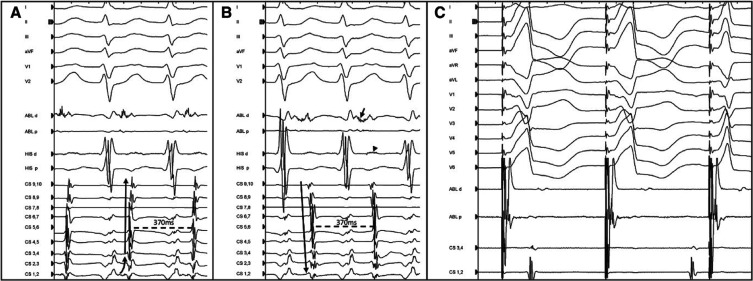
Endocardial tracings during orthodromic atrioventricular tachycardia in case 2. The ablation catheter is located at the posterolateral mitral annulus recording a short V–A interval and a fractionated atrial electrogram **(A)**. After an application of RF energy at this position, the atrial sequence along the coronary sinus catheter is reversed without change in the tachycardia cycle length. The V–A interval is shorter in the distal ablation bipole, which is positioned distally to the coronary sinus catheter, than the one recorded at the His catheter (arrowhead) suggesting an intra-atrial block and counterclockwise retrograde atrial activation **(B)**. **C:** Postablation V–A dissociation during ventricular pacing, which confirms the successful accessory pathway elimination after radiofrequency energy application at the lateral atrioventricular annulus.

A 52-year-old man with a history of previous CA of a concealed, left lateral AP and recurrent supraventricular tachycardia was referred for a redo CA. The presence of a free-wall AP was confirmed by ventricular pacing, which demonstrated non-decremental conduction with eccentric atrial activation and a relatively long V–A interval. During ventricular pacing, an ORT was induced. Mapping of the AP was performed after gaining access in the left atrium (LA). At a posterolateral site of the mitral annulus, a very short V–A interval with a fractionated atrial electrogram was recorded, suggesting a good target site for ablation **(Figure 2A)**. Following RF energy delivery at this point, the atrial activation changed to a concentric sequence without any effect on the TCL. Despite the concentric atrial activation sequence in the CS, the ablation catheter recorded continuous V–A activation at the lateral mitral annulus, which was earlier than the atrial activation recorded by the His catheter **(Figure 2B)**. Additional RF application was delivered in a more lateral position (3 o’clock in the LAO 40° projection) compared to the previous lesion, resulting in the termination of the tachycardia. Following ablation, the tachycardia was no longer inducible, while ventricular pacing demonstrated a ventriculoatrial dissociation, thus confirming the successful elimination of the AP **(Figure 2C)**.

## Discussion

One of the important clues in the localization of an AP during an ORT is the analysis of the retrograde atrial activation sequence. An ORT with an eccentric atrial activation sequence rules out the participation of a right, septal or posteroseptal AP, while a concentric CS activation pattern usually excludes the participation of a left AP in the tachycardia circuit. However, in the case of a narrow QRS tachycardia with an eccentric retrograde activation sequence, electrophysiology testing and specific maneuvers are needed to rule out atrial tachycardia and atypical AVNRT.^5^ Atypical AVNRT with an eccentric retrograde left-sided activation can be present in 6% of patients with AVNRT, which needs to be differentiated from an ORT using a left-sided AP.^6^ A possible explanation of the eccentric retrograde activation sequence during AVNRT could also be the involvement of the ligament of Marshall as part of the circuit.^7^ The presence of a long PPI–TCL during tachycardia and the presence of decremental properties strongly support the diagnosis of an atypical AVNRT. In our cases, corrected PPI–TCL was <110 ms and the SA–VA difference was <85 ms during tachycardia, and non-decremental conduction properties during ventricular pacing were observed, ruling out AVNRT.

**Figure 3:**
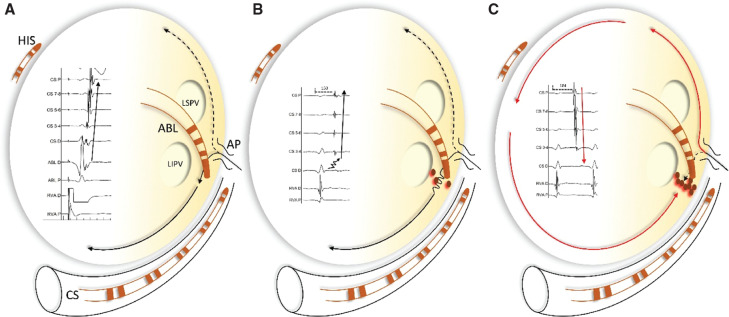
Schematic representation of an intra-atrial conduction block during radiofrequency (RF) ablation of left lateral accessory pathway. **A**: A left lateral accessory pathway and a distal to proximal coronary sinus activation. **B**: After RF energy delivery at the level of the mitral isthmus, the coronary sinus is still activated from distal to proximal but the V–A interval is prolonged due to partial intra-atrial block. **C**: Further RF lesions lead to complete intra-atrial conduction block and to counterclockwise atrial activation.

We report these 2 cases of ORT via a left-sided AP with an intraprocedural change in retrograde atrial activation during RF energy delivery, from an eccentric to a concentric sequence. A possible mechanism of this phenomenon is the occurrence of an intra-atrial conduction block at the mitral isthmus level medial to the insertion of the AP. As a result, there is still retrograde atrial activation via the AP, but with intra-atrial impulse propagation in a counterclockwise direction, thus depolarizing the inferior LA in a concentric sequence. The above-described propagation of the depolarization wavefront, in the context of the underlying intra-atrial block, explains the change in CS activation from an eccentric to a concentric pattern during tachycardia, with delayed activation of the proximal CS compared to the lateral mitral annulus distally to the level of the block **(Figure 3)**.

Familiarity with the likelihood of an intra-atrial block during left lateral AP ablation is needed to avoid erroneous elucidation that a second AP is present. Assessment of relative conduction time proximal and distal to the ablation level is helpful to elucidate the direction of propagation wavefront during tachycardia.

Luria et al. reported that intra-atrial conduction block may occur in 6.9% of left free-wall AP cases following RF delivery along the mitral isthmus, which anatomically is the narrower conductive tissue between the mitral annulus and the left inferior pulmonary vein.^2^ However, intra-atrial conduction block can occur even in a wide mitral isthmus, possibly because of areas of pre-existing functional block or scar.^8^

Rarely, the presence of an AP with multiple bundles connecting the ventricle to the atrium at a lateral but also more posterior portions of the mitral annulus could be another explanation of this phenomenon.^9^ Once the RF lesion eliminates the lateral atrial insertion of the AP, the atrial activation may be modified from an eccentric to a concentric or midline pattern if a second atrial limb of the AP connects to the proximal CS. However, if this hypothesis held true in our cases, we would expect a probable change in the TCL and a later retrograde atrial activation in the lateral as compared to the posterior mitral annulus. Finally, it is worth noting that bidirectional intra-atrial conduction block can occur after an unsuccessful endocardial ablation of APs, which may have a subepicardial or an epicardial course requiring an epicardial ablation from the CS.^10^

## Conclusion

An intra-atrial conduction block at the level of the mitral isthmus may occur during the CA of left-sided APs. Complete or partial intra-atrial block should be suspected when an abrupt change in the atrial activation sequence is noted during CA at the posterolateral and lateral aspects of the mitral annulus. Recognition of this phenomenon is necessary for the accurate location of the site of interest and successful AP ablation while avoiding unnecessary lesion deployment.
